# Association between brain volume and depression in Alzheimer's disease: Neuroimaging insights

**DOI:** 10.1002/alz.71120

**Published:** 2026-01-29

**Authors:** Chao Tang, Jiaxin Yang, Xiaoyang Lei, Ming Zhang, Yi Chen, Xiaoxue Peng, Dian He

**Affiliations:** ^1^ Department of Neurology Affiliated Hospital of Guizhou Medical University Guiyang Guizhou Province China

**Keywords:** Alzheimer's disease, cognitive impairment, depression, vascular depression, white matter hyperintensities

## Abstract

**INTRODUCTION:**

Alzheimer's disease (AD) often co‐occurs with depression, affecting cognitive function and quality of life. Understanding the neurobiological links between brain abnormalities and depressive symptoms is essential for effective treatment.

**METHODS:**

We analyzed 2,722 participants from the National Alzheimer's Coordinating Center, including 886 AD patients and 1,836 cognitively normal controls. Neuroimaging assessed brain volumes, while depressive symptoms were measured using the Geriatric Depression Scale. Multiple linear regression and mediation analyses evaluated associations between brain structure, cognitive function, and depression.

**RESULTS:**

AD patients had significantly higher rates of depressive symptoms (35.3% vs. 14.7%; *p* < 0.001) and cognitive impairments (mean Mini‐Mental State Examination [MMSE]: 23.1 vs. 28.9; *p* < 0.001). Hippocampal atrophy mediated the relationship between depression and AD (indirect effect = −0.107; *p* < 0.001).

**CONCLUSION:**

Hippocampal atrophy significantly mediates the relationship between depression and AD, suggesting targeted interventions may enhance patient outcomes.

## Highlights

- Alzheimer's disease (AD) patients show significant hippocampal atrophy linked to depression.
- Reduced hippocampal volume correlates strongly with cognitive impairment severity.
- Depression occurrence is higher in AD, impacting overall patient quality of life.
- Neurological pathways connecting depression and brain changes warrant further study.
- Targeted interventions may improve cognitive and emotional health in AD patients.

## 1 BACKGROUND

Alzheimer's disease (AD) is a progressive neurodegenerative disorder characterized by cognitive decline and impairments in daily functioning.^1^ It presents a significant global health challenge, with the prevalence expected to increase dramatically; the World Health Organization estimates that approximately 50 million people worldwide currently live with dementia, projected to rise to 152 million by 2050.^2^ The complex pathophysiology of AD involves amyloid‐beta accumulation, tau protein hyperphosphorylation, and neuroinflammation, but the interplay between AD and mood disorders, particularly depression, remains underexplored.^3^

Depression frequently co‐occurs with AD, significantly impacting quality of life and exacerbating cognitive decline. Studies indicate that between 20% and 50% of individuals with AD may experience depressive symptoms, complicating both diagnosis and treatment.^4^, ^5^ These symptoms manifest as psychological issues and critical factors that accelerate cognitive impairment. Research shows that depressive symptoms, including anhedonia, feelings of worthlessness, fatigue, and cognitive dysfunction, often overlap with AD‐related cognitive deficits.^6^ This overlap complicates clinical assessments, making it challenging for healthcare providers to differentiate cognitive decline due to AD from that resulting from depression.^7^

The relationship between depression and AD is bidirectional. Cognitive decline may lead to feelings of helplessness and despair, contributing to depressive symptoms. Conversely, depression is a risk factor for cognitive decline, potentially initiating or accelerating neurodegeneration associated with AD.^8^ This complex interaction underscores the need for integrated treatment approaches that address both cognitive and emotional aspects.^9^ Understanding this interrelation is crucial for effective patient management and the development of comprehensive treatment strategies, including pharmacological and non‐pharmacological interventions.^10^

Recent neuroimaging studies have highlighted the neuroanatomical correlates of AD and depression, utilizing advanced techniques like magnetic resonance imaging (MRI).^11^ These scans allow for quantifying brain structures, enabling assessments of neuroanatomical changes associated with both conditions. Hippocampal atrophy is a well‐documented hallmark of AD, crucial for memory formation.^12^ Furthermore, studies have shown that patients with both AD and depression exhibit additional volume reductions in regions such as the frontal cortex, which plays a key role in mood regulation. Alterations in white matter integrity and hyperintensities have also been linked to cognitive and emotional dysfunctions in both conditions.^13^, ^14^, ^15^

A recent meta‐analysis demonstrated that geriatric depression significantly predicts future AD diagnosis, with an odds ratio of 1.28, indicating a heightened risk of AD among older adults with depressive symptoms.^16^ This temporal relationship supports the hypothesis that depression may not merely be a consequence of neurodegeneration in AD but could also contribute to AD pathology through mechanisms such as neuroinflammation and hippocampal atrophy.^13^, ^17^

Research shows that certain neuroanatomical changes, such as reductions in hippocampal volume and increases in lateral ventricular size, may predict depressive symptoms in AD patients.^18^ Neuroanatomical abnormalities, including white matter lesions, correlate with the severity of depressive symptoms, suggesting these brain changes may underpin overlapping cognitive and emotional deficits.^13^, ^19^ Understanding the neurobiological foundations of these relationships enhances insights into how AD and depression coexist, informing more effective interventions for affected individuals.

Despite progress, a critical gap remains in understanding the neurobiological substrates linking AD and depression. A key question is whether depressive symptoms in AD patients merely reflect overlapping cognitive disturbances or indicate independent pathophysiological processes exacerbating cognitive deficits.^20^ Increasing evidence suggests that late‐life depression may independently contribute to cognitive decline through distinct mechanisms, such as accelerated hippocampal atrophy.^21^

To address this gap, we systematically evaluated the prevalence of depressive symptoms in AD patients compared to cognitively intact controls using data from the National Alzheimer's Coordinating Center (NACC). We hypothesize that depression may operate through both “vascular” and “neurodegenerative” mechanisms, with neuroimaging markers of brain structural changes mediating the depression‐cognition relationship. By integrating comprehensive neuropsychological assessments with structural MRI‐derived measurements, we aim to elucidate how specific brain volume abnormalities‐particularly in the hippocampus and frontal cortex‐correlate with depression severity and cognitive deficits in AD, informing targeted interventions to enhance clinical outcomes.

## 2 METHODS

### 2.1 Study population

This study utilized comprehensive data from the NACC Uniform Data Set, which includes clinical assessments, neuroimaging data, and genetic information.^22^, ^23^ The selected sample comprised 2722 participants, including 886 individuals diagnosed with AD and 1836 cognitively normal control subjects. The aim of this research is to explore the comorbidity of AD and depression while examining the impact of abnormal brain volumes on these associations.

Participants' demographic information includes age (in years), sex (male/female), race (Caucasian, African American, Hispanic, other), and years of education, providing a foundational basis for analyzing the interactions between AD, depression, and brain volume abnormalities. All sample data can be found in the Table S1.

**RESEARCH IN CONTEXT**

1. **Systematic review**: We conducted a thorough review of literature on the neurobiological correlations between brain volume abnormalities and depression in Alzheimer's Disease (AD). Our analysis revealed significant associations between hippocampal atrophy, cortical thinning, and ventricular enlargement with both cognitive impairment and elevated depressive symptoms. These findings highlight critical gaps in understanding the interplay between structural brain changes and mood disorders in AD.
2. **Interpretation**: Our results indicate that AD patients exhibit notable reductions in hippocampal volume, which are strongly correlated with severity of depressive symptoms. The robust evidence suggests that hippocampal atrophy may be a key neurobiological mediator, emphasizing the dual impact of cognitive decline and depression on patient outcomes.
3. **Future directions**: Key research questions have emerged, including the need to explore the role of neuroinflammation and vascular health in brain volume abnormalities related to depressive symptoms. Future studies utilizing longitudinal designs and advanced neuroimaging techniques are essential to unravel these complex relationships and inform targeted interventions addressing both cognitive and emotional health.

### 2.2 Inclusion and exclusion criteria

#### 2.2.1 Diagnostic groups and inclusion criteria

Participants were included in the study if they met the following criteria:

Cognitively normal control group:

The cognitively normal control group was defined as individuals with a clinical diagnosis of “cognitively normal” according to NACC criteria, meeting all of the following requirements:

1. Age of 55 years or older.
2. Mini‐Mental State Examination (MMSE) scores ≥ 27 (out of 30).
3. Clinical Dementia Rating (CDR) global score of 0.
4. Individuals diagnosed with mild cognitive impairment (MCI) were explicitly excluded to ensure a clear cognitive distinction between AD patients and controls.
5. Completion of necessary cognitive assessments and neuroimaging protocols, ensuring that adequate data was available for analysis.

Alzheimer's disease group:

The AD group consisted of participants with a clinical diagnosis of probable or possible AD according to National Institute of Neurological and Communicative Disorders and Stroke‐Alzheimer's Disease and Related Disorders Association (NINCDS‐ADRDA) criteria, meeting all of the following requirements:

1. Age of 55 years or older.
2. MMSE scores≤ 24.
3. CDR global scores≥ 1.
4. Comprehensive clinical evaluations following guidelines from the National Institute on Aging‐Alzheimer's Association (NIA‐AA).
5. Completion of necessary cognitive assessments and neuroimaging protocols, ensuring that adequate data was available for analysis.

To account for residual variability in cognitive functioning within diagnostic groups, MMSE scores were included as a covariate in all regression models examining associations between brain structural measures and depressive symptoms.

#### 2.2.2 Exclusion criteria

Individuals were excluded from the study if they had:

1. A history or concurrent presence of significant neurodegenerative diseases other than AD (e.g., frontotemporal dementia, vascular dementia, or Lewy body dementia).
2. Severe medical complications or comorbid psychiatric conditions that could confound cognitive testing outcomes (e.g., untreated schizophrenia or bipolar disorder). Note that depressive symptoms were not exclusionary, as depression represented a key variable of interest in this study.
3. Incomplete data for critical variables necessary for robust statistical analysis, particularly cognitive assessments (Geriatric Depression Scale [GDS], MMSE, CDR) or neuroimaging results MRI ‐derived volumetric measures).

These criteria were established to ensure that the selected population maintained diagnostic homogeneity while providing adequate cognitive separation between groups, thereby reducing potential confounding influences on the outcomes of interest.

### 2.3 Variables measurement and definitions

#### 2.3.1 Neuropsychological assessments

In this study, several neuropsychological assessments were employed to evaluate cognitive function and depressive symptoms among participants. The Global CDR Score measures the stage of dementia, while the CDR Sum Score provides a composite score from multiple cognitive assessments. The MMSE Score assesses cognitive functions such as attention, memory, and language. Language abilities are further evaluated through the Boston Naming Test Score. The Logical Memory Score assesses episodic memory, and the Memory Units Recalled indicate the number of items remembered during this test. The Digit Span Forward Trials Correct and Digit Span Length measure working memory capacity, whereas the Digit Span Backward Trials Correct and Digit Span Length Backwards evaluate backward recall abilities. Participants’ verbal fluency is documented by the number of animals and vegetables they can name within a specific timeframe. Additionally, the Trail Making Test Part A Score assesses attention and task‐switching abilities. To evaluate mood symptoms, the GDS is used, with the GDS Score reflecting the overall level of depressive symptoms in older adults. These variables collectively provide vital information for evaluating cognitive ability and emotional state, constituting the foundation for subsequent analyses.

#### 2.3.2 Brain volume measurements

Structural MRI scans were utilized to quantify various brain structures, which are crucial for understanding the neuroanatomical correlates of AD and depression. Key measurements included ADC Volume, indicating cellular density or edema status; Intracranial Volume, representing the overall size of the brain and surrounding tissues; and  brain atrophy volume, which refers to the volume of removed brain tissue. Additionally, cerebrospinal fluid (CSF) Volume acts as a marker for brain atrophy, while Gray matter volume and White matter volume reflect the density of neuronal cell bodies and the integrity of white matter fiber tracts, respectively. The White Matter Hyperintensity Volume may be associated with small vessel disease or other pathological processes. Combined, Cerebral Total Volume represents both gray and white matter volumes, and Hippocampal Volume is critical for memory function. Other crucial measures include Cerebral CSF Volume, Cerebral Gray matter volume, Cerebral White matter volume, Left and Right Hippocampal Volumes, and volumes of the Left and Right Lateral Ventricles. The Total lateral ventricular volume, along with the volumes of the Thalamus, Left and Right Frontal Cortexes, Total Frontal Cortex, Left and Right Occipital Cortexes, and Parietal Cortex, provides a comprehensive assessment of brain structure. These Brain Volume measurements serve as essential data points for investigating the relationships between neuroanatomical changes, cognitive deficits, and depressive symptoms.

#### 2.3.3 Neuroimaging acquisition and processing

Given the multi‐site nature of the NACC database, neuroimaging data were acquired across multiple ADRCs using varying MRI protocols. To address the technical heterogeneity inherent in multi‐site data collection, we implemented comprehensive quality control and harmonization procedures. Structural MRI scans were acquired using 1.5T and 3.0T scanners across participating sites, with predominant sequences including T1‐weighted 3D magnetization‐prepared rapid gradient‐echo (MPRAGE) acquisitions with typical parameters: repetition time (TR) = 2300‐3000 ms, echo time (TE) = 2.8‐3.5 ms, flip angle = 8‐9°, field of view (FOV) = 240‐256 mm, and voxel size = 1×1×1 mm^3^. T2‐weighted fluid‐attenuated inversion recovery (FLAIR) sequences were also obtained for white matter hyperintensity assessment. All structural MRI scans underwent standardized processing using FreeSurfer version 6.0 (Laboratory for Computational Neuroimaging, Athinoula A. Martinos Center for Biomedical Imaging, Boston, MA) and FSL version 6.0 (FMRIB Software Library, Oxford, UK), with automated segmentation algorithms applied to quantify regional brain volumes, including hippocampal volumes, cortical thickness measurements, ventricular volumes, and white matter hyperintensity burden. Quality assurance procedures included visual inspection of segmentation outputs and exclusion of scans with significant motion artifacts or processing failures. To account for potential scanner and site‐related variability, we employed multiple strategies: (1) scanner field strength (1.5T vs. 3.0T) and acquisition site were included as covariates in all multivariable regression and machine learning (ML) models to statistically control for technical variance; (2) the ComBat harmonization method (using the R package ‘neuroCombat’) was applied to volumetric measurements to remove scanner effects while preserving biological variability, using an empirical Bayes approach to adjust for additive and multiplicative site effects; (3) all regional brain volumes were normalized to total intracranial volume (ICV) to account for individual differences in head size and scanner‐related scaling effects, using the formula: normalized volume = (regional volume / ICV)× mean ICV across all participants; and (4) sensitivity analyses were conducted stratified by scanner field strength, with tests for interaction effects between imaging parameters and clinical variables to ensure that observed associations between brain volumes, depression, and AD were not driven by acquisition heterogeneity. These preprocessing and harmonization procedures aimed to minimize the potential confounding effects of multi‐site acquisition variability while preserving the biological signals of interest related to neurodegenerative and neuropsychiatric processes.

### 2.4 Data organization and preprocessing

Data extraction and preprocessing were meticulously conducted, ensuring clarity and organization according to best practices:

1. Data Cleaning: Initial steps involved scrutinizing the dataset for inconsistencies, missing values, and outliers. Cleaning of incomplete records was performed to enhance the robustness of statistical analyses.
2. Normality Assessment: Normal distribution of continuous variables (e.g., MMSE scores, GDS scores) was assessed using Shapiro–Wilk tests. This guided the choice of statistical methods for further comparisons, whether parametric or non‐parametric.
3. Handling Missing Data: Multiple imputation techniques were employed for managing missing values using the MICE (Multivariate Imputation by Chained Equations) package in R. Predictive mean matching (PMM) was used for continuous variables, and logistic regression for binary variables. Five imputed datasets were generated (*m* = 5), and results were pooled using Rubin's rules. Variables with > 20% missing data were excluded from primary analyses. The proportion of missing data for key variables ranged from 2.3% (age, sex) to 15.7% (specific neuroimaging measures), with a detailed missingness pattern analysis conducted to assess whether data were missing completely at random (MCAR), missing at random (MAR), or missing not at random (MNAR) using Little's MCAR test. These preprocessing methods ensured that the dataset was adequately prepared for subsequent statistical analyses while minimizing bias introduced by missing data.

These preprocessing methods ensured that the dataset was adequately prepared for subsequent statistical analyses.

### 2.5 Statistical methods

All statistical analyses were conducted using R version 4.3.1, employing various specialized packages for rigorous data manipulation and evaluation. Key elements of statistical analysis included:

#### 2.5.1 Descriptive statistics

Descriptive analyses provided comprehensive summaries of demographic and clinical characteristics for both groups (AD and controls). Continuous variables were represented as means and standard deviations (SDs), while categorical variables were expressed as frequencies and percentages. Normality tests (Shapiro–Wilk) were conducted on continuous variables to inform the selection of analyses.

#### 2.5.2 Group comparisons

Comparative analyses between AD patients and control participants utilized the following statistical tests:

*t‐Tests*: For normally distributed continuous variables (e.g., age, MMSE scores).
*Mann–Whitney U Tests*: For non‐normally distributed metrics, particularly GDS scores, to provide insights into central tendency differences.
*Chi‐Squared Tests*: For analyzing differences between groups regarding demographic and categorical variables, like the presence of the apolipoprotein E (APOE) ε4 allele.
*Effect Sizes* were calculated to quantify the magnitude of group differences: Cohen's d for continuous variables (with *d* = 0.2, 0.5, and 0.8 interpreted as small, medium, and large effects, respectively), Cramér's V for categorical variables, and rank‐biserial correlation for non‐parametric tests. All effect sizes are reported alongside *p*‐values to facilitate clinical interpretation beyond statistical significance.

All statistical tests employed a two‐tailed approach, with a significance level set at *p* < 0.05.

#### 2.5.3 Regression analysis

To assess the relationships between brain volumes and depression severity in the context of AD, multiple regression analyses were employed:

*Multiple Linear Regression Models*: These models evaluated the impact of brain volumetric measures (e.g., hippocampal volume) on GDS scores, controlling for confounding factors such as age, sex, and education level. Model diagnostics included checks for homoscedasticity, normality of residuals, and multicollinearity (using variance inflation factor, VIF).

#### 2.5.4 ML analyses

We employed ML approaches to complement traditional statistical analyses, addressing distinct research objectives. While parametric statistical analyses were used for hypothesis testing and causal inference (determining whether specific brain regions are associated with depressive symptoms and AD, and estimating effect sizes), ML analyses focused on multivariate prediction for potential clinical decision support. Specifically, we developed separate predictive models to: (1) classify AD diagnosis (binary classification: AD vs. cognitively normal) using combined neuroimaging, demographic, and clinical features; and (2) predict depression severity (GDS total score as continuous outcome) from neuroimaging and clinical markers. Unlike statistical models that assume linear relationships and test individual predictors, ML models capture complex non‐linear interactions among multiple features and prioritize predictive accuracy. These complementary approaches address both mechanistic understanding (statistical analyses) and clinical prediction (ML analyses).

To ensure robust and generalizable models while minimizing overfitting and Type I error inflation, we restricted candidate features to a theoretically‐driven set based on established neurobiology of depression and AD (*n* = 12 total features): bilateral hippocampal volumes, bilateral entorhinal cortex thickness, precuneus cortical thickness, posterior cingulate cortex volume, total white matter hyperintensity burden, ventricular volume, age, sex, education years, and APOEε4 carrier status. We explicitly avoided automated stepwise feature selection procedures that could capitalize on chance fluctuations, instead using domain knowledge to constrain the feature space and reduce risk of spurious associations. All continuous features were standardized (z‐scored) prior to model training.

For both prediction tasks, we compared three algorithms: Random Forest (RF), Support Vector Machine with radial basis function kernel (SVM), and XGBoost. Data were split into training (70%), validation (15%), and held‐out test sets (15%) using stratified sampling to maintain outcome distribution. Model development employed nested cross‐validation: the outer loop consisted of 10‐fold cross‐validation for unbiased performance evaluation, while the inner loop used 5‐fold cross‐validation for hyperparameter tuning. For AD classification, we evaluated models using area under the receiver operating characteristic curve (AUC‐ROC) as the primary metric, with secondary metrics including sensitivity, specificity, positive predictive value (PPV), negative predictive value (NPV), F1‐score, and calibration plots. For depression severity prediction (regression task), we used mean absolute error (MAE) as the primary metric, with secondary metrics including root mean squared error (RMSE), R^2^, and explained variance.

Statistical significance of model performance was assessed through permutation testing (*n* = 1,000 permutations) to establish empirical null distributions rather than using inappropriate parametric alpha thresholds. Given that we conducted two distinct ML prediction tasks with three algorithms each, we applied hierarchical Bonferroni correction: an overall correction for two tasks (adjusted *α* = 0.05/2 = 0.025), and within‐task correction for three algorithms (adjusted *α* = 0.025/3 = 0.008). Models were considered significantly better than chance only if permutation‐based *p *< 0.008.

To assess model stability, we repeated the entire nested cross‐validation procedure 100 times with different random seeds and calculated the coefficient of variation (CV) for performance metrics. Models with CV > 0.15 were deemed unstable and excluded. We derived 95% confidence intervals for all performance metrics through bootstrapping (1,000 iterations). Feature importance was quantified using SHAP (SHapley Additive exPlanations) values for the best‐performing model in each task. Features were considered reliably important only if they ranked in the top 5 contributors in ≥80% of the 100 repeated CV runs. Additional safeguards against overfitting included early stopping during model training, regularization (L1/L2 penalties), maximum tree depth constraints, and examination of learning curves. Final model performance was always reported on the held‐out test set that was never exposed during model development or hyperparameter tuning.

#### 2.5.5 Mediation analysis

Given the increased Type I error risk associated with mediation analyses, particularly when preceded by multiple statistical tests, we implemented a hierarchical analytical strategy that distinguishes between confirmatory hypothesis‐driven analyses and exploratory hypothesis‐generating analyses, each with appropriate statistical safeguards. Our primary hypothesis, grounded in the“vascular depression” hypothesis, posits that hippocampal atrophy mediates the relationship between baseline depressive symptoms (GDS total score) and AD diagnosis.

Mediation analyses followed a causal steps approach supported by formal tests of indirect effects, ensuring that prerequisite conditions were met: (a) the independent variable (GDS score) was significantly associated with the dependent variable (AD diagnosis); (b) the GDS score was significantly linked to the mediator (hippocampal volume); and (c) the mediator (hippocampal volume) was significantly correlated with the dependent variable (AD diagnosis) while controlling for the independent variable. We tested three pre‐specified mediators: left hippocampal volume, right hippocampal volume, and mean bilateral hippocampal volume (*n* = 3 mediation models), all normalized to intracranial volume.

To explore the temporal directionality of the depression‐hippocampal atrophy‐AD relationship, we assessed bidirectional mediation pathways: Path 1 (Depression → Hippocampus → AD) and Path 2 (AD → Hippocampus → Depression). Indirect effects were estimated using bias‐corrected bootstrap confidence intervals with 10,000 samples, controlling for Type I error inflation through Bonferroni correction (adjusted *α* = 0.0083 for six tests). Indirect effects were considered statistically significant only if the 99.17% CI excluded zero.

Confounders including age, sex, education, APOE ε4 status, MMSE scores, scanner field strength, and acquisition site were controlled. For exploratory post‐hoc analyses, we examined 18 additional brain volumetric measures, testing the same bidirectional pathways. These exploratory analyses were subjected to false discovery rate (FDR) correction (Benjamini‐Hochberg procedure, *q* < 0.05), with results surviving correction reported as potentially significant exploratory findings.

#### 2.5.6 Primary and secondary outcomes

To address concerns regarding multiple comparisons and control for Type I error inflation, we explicitly distinguished between primary (confirmatory) and secondary (exploratory) analyses. Primary analyses focused on a priori hypothesized brain regions based on existing literature linking depression and AD pathology, specifically bilateral hippocampal volumes and entorhinal cortex thickness. For these primary outcomes, a significance threshold of *α* = 0.05 (two‐tailed) was applied without correction, as these tests were hypothesis‐driven and limited in number (*n* = 2‐3 primary comparisons). Secondary exploratory analyses examined additional brain regions and neuroimaging markers, including frontal and temporal cortical volumes, ventricular volumes, and white matter hyperintensity burden. For these secondary analyses involving multiple comparisons (*n* = 15‐20 tests), we applied the FDR correction using the Benjamini–Hochberg procedure to control for Type I error, with a corrected significance threshold of *q* < 0.05. Results from exploratory analyses were interpreted with appropriate caution and clearly labeled as hypothesis‐generating rather than confirmatory findings.

### 2.6 Software

This study utilized R software version 4.3.0 for statistical analyses. Key R packages implemented included mediation for conducting mediation analyses, mice for handling missing data through multiple imputation, and *E* Value for sensitivity analyses to assess robustness against unmeasured confounding. Additionally, we applied the p. adjust function for FDR correction to control for multiple comparisons. Advanced visualizations were facilitated through various plotting libraries, ensuring comprehensive data presentation and analysis.

### 2.7 Ethical considerations

This study utilized de‐identified data sourced from the NACC database. Written informed consent was obtained from participants at each ADRC, with ethical approval granted by each institution's review board. Since the analysis utilized a de‐identified dataset, Institutional Review Board (IRB) approval was deemed unnecessary according to federal regulations. Data access adhered to strict data use agreements, ensuring comprehensive privacy protections and confidentiality through established anonymization protocols within the NACC.

## 3 RESULTS

### 3.1 Demographics and clinical characteristics of the study population

In this study, we examined a total of 2722 participants, consisting of 1836 individuals in the control group and 886 individuals diagnosed with AD (Table 1). A significant gender imbalance was observed, with a higher proportion of males in the AD group (52.37% vs. 33.61% in the control group, χ2 = 87.8891, *p* < 0.001). The mean age of participants was significantly higher in the AD group (76.87 ± 9.03 years) compared to the control group (71.43 ± 10.47 years, *t* = −13.9692, *p* < 0.001). While both groups had similar years of education (15.35 ± 3.43 years overall), the control group averaged slightly more years of education (15.50 ± 3.32 years) compared to the AD group (15.05 ± 3.62 years, *t* = 3.1232, *p* = 0.002).

**TABLE 1:** Demographic and cognitive characteristics of participants.

Variables	Total (*n* = 2722)	Control (*n* = 1836)	AD (*n* = 886)	Statistics	*p*‐value
Gender, *n* (%)				*χ* ^2^ = 87.89	<0.001
Male	1,081 (39.71)	617 (33.61)	464 (52.37)		
Female	1,641 (60.29)	1,219 (66.39)	422 (47.63)		
Age, mean ± SD (years)	73.20 ± 10.34	71.43 ± 10.47	76.87 ± 9.03	*t* = −13.97	<0.001
Years of education, mean ± SD	15.35 ± 3.43	15.50 ± 3.32	15.05 ± 3.62	*t* = 3.12	0.002
APOE, n (%)				*Z* = −10.94	<0.001
0	1,670 (61.35)	1,244 (67.76)	426 (48.08)		
1	905 (33.25)	547 (29.79)	358 (40.41)		
2	147 (5.40)	45 (2.45)	102 (11.51)		
GDS, n (%)				*χ* ^2^ = 54.92	<0.001
None	2,680 (98.46)	1,830 (99.67)	850 (95.94)		
Yes	42 (1.54)	6 (0.33)	36 (4.06)		
GDS score, M (Q1, Q3)	1.00 (0.00, 2.00)	1.00 (0.00, 2.00)	1.00 (1.00, 3.00)	*Z* = −14.24	<0.001
Depression, n (%)				*χ* ^2^ = 150.99	<0.001
Yes	583 (21.42)	270 (14.71)	313 (35.33)		
None	2,139 (78.58)	1,566 (85.29)	573 (64.67)		
Severity of depression, *n* (%)				*χ* ^2^ = 156.12	<0.001
Moderate	155 (5.69)	69 (3.76)	86 (9.71)		
None	2,139 (78.58)	1,566 (85.29)	573 (64.67)		
Mild	392 (14.40)	190 (10.35)	202 (22.80)		
Severe	36 (1.32)	11 (0.60)	25 (2.82)		
CDR‐S, M (Q1, Q3)	0.00 (0.00, 1.50)	0.50 (0.20, 1.60)	3.50 (1.50, 5.50)	*Z* = −43.66	<0.001
MMSE Score, Mean ± SD	27.01 ± 4.32	28.89 ± 1.46	23.12 ± 5.51	*t* = 30.65	<0.001
Boston Naming Test Score, Mean ± SD	25.49 ± 5.45	27.49 ± 3.04	21.34 ± 6.83	*t* = 25.59	<0.001
Logical Memory Score, M (Q1, Q3)	12.00 (7.00, 16.00)	14.00 (11.00, 17.00)	5.00 (3.00, 9.00)	*Z* = 33.77	<0.001
Memory Units Recalled, M (Q1, Q3)	10.00 (5.00, 15.00)	13.00 (10.00, 16.00)	2.00 (0.00, 6.00)	*Z* = 35.83	<0.001
Digit Span Forward Trials Correct, Mean ± SD	8.06 ± 2.18	8.37 ± 2.06	7.41 ± 2.27	*t* = 11.10	<0.001
Digit Span Length, Mean ± SD	6.35 ± 1.30	6.52 ± 1.19	5.98 ± 1.42	*t* = 10.34	<0.001
Digit Span Backward Trials Correct, M (Q1, Q3)	6.00 (5.00, 8.00)	6.00 (5.00, 8.00)	5.00 (4.00, 7.00)	*Z* = 14.73	<0.001
Digit Span Length Backwards, M (Q1, Q3)	4.00 (4.00, 5.00)	5.00 (4.00, 6.00)	4.00 (3.00, 5.00)	*Z* = 14.40	<0.001
Animals, M (Q1, Q3)	18.00 (14.00, 23.00)	21.00 (17.00, 25.00)	12.00 (9.00, 16.00)	*Z* = 31.00	<0.001
Vegetables, M (Q1, Q3)	13.00 (9.00, 16.00)	15.00 (12.00, 17.00)	8.00 (5.00, 10.00)	*Z* = 31.91	<0.001
Trail Making Test Part A Score, M (Q1, Q3)	33.00 (24.00, 47.00)	29.00 (22.00, 38.00)	47.00 (33.00, 72.00)	*Z* = −24.09	<0.001

*Note*: Data are presented as n(%) for categorical variables and as mean ± standard deviation for continuous variables. The *p*‐values were adjusted for multiple comparisons using the false discovery rate (FDR) method.

Abbreviations: AD = Alzheimer's disease; ADC = apparent diffusion coefficient; APOE, apolipoprotein E; CDR, Clinical Dementia Rating; GDS, Geriatric Depression Scale; MMSE, Mini‐Mental State Examination.

The distribution of APOE genotypes showed notable differences between groups (*Z* = −10.9413, p < 0.001); the frequency of APOEε2 alleles was significantly lower in the AD group (2.45%) compared to the control group (67.76%), while the proportion of APOE ε4 alleles was significantly higher in the AD group (40.41% vs. 33.25%).

Regarding depressive symptoms, assessment using the GDS revealed significant between‐group differences. Among the total sample, 21.4% (*n* = 583) met criteria for clinically significant depressive symptoms (GDS ≥ 5), with substantially higher prevalence in the AD group (35.3%, *n* = 313) compared to controls (14.7%, *n* = 270; *χ* ^2^ = 150.99, *p* < 0.001). The median GDS score was significantly higher in the AD group (Median = 1.0, interquartile range [IQR]: 1.0–3.0) compared to controls (Median = 1.0, IQR: 0.0–2.0; *Z* = −14.24, *p* < 0.001). Depression severity distribution also differed markedly between groups (*χ* ^2^ = 156.12, p < 0.001), with higher rates of moderate (9.7% vs. 3.8%) and severe depression (2.8% vs. 0.6%) in the AD cohort.

Cognitive assessments yielded statistically significant findings, notably in the CDR Sum Score, which was significantly higher in the AD group (*M* = 3.50) compared to the controls (*M* = 0.50, *Z* = −43.6603, *p* < 0.001). The MMSE scores indicated that the AD group (23.12 ± 5.51) performed significantly worse than the control group (28.89 ± 1.46, *t* = 30.6462, *p* < 0.001). Additional cognitive tests such as the Boston Naming Test and Logical Memory test also demonstrated lower scores in the AD group, indicating impaired cognitive function.

### 3.2 Neuroimaging and cerebral volume characteristics of the study population

In this study, we evaluated the neuroimaging and cerebral volume characteristics of a total of 2722 participants, which included 1836 individuals in the control group and 886 individuals diagnosed with AD (Table 2). The comparison of the apparent diffusion coefficient (ADC) volume showed no significant difference between the two groups, with medians of 6499.000 across both groups (*Z* = −7.1673, *p* < 0.001). In contrast, the brain atrophy volume was significantly lower in the AD group (998.772 ± 107.814) compared to controls (1037.867 ± 118.791, *t* = 8.5712, *p* < 0.001).

**TABLE 2:** Neuroimaging and cerebral volume characteristics of the study population.

Variables	Total (n = 2722)	Control (*n* = 1836)	AD (*n* = 886)	Statistics	*p*‐value
ADC volume, M (Q1, Q3)	6499.00 (6061.00, 6518.00)	6499.00 (6061.00, 6518.00)	6518.00 (6061.00, 6518.00)	*Z* = −7.17	<0.001
Intracranial volume, mean ± SD (cm^3^)	1379.62 ± 146.69	1376.37 ± 146.47	1386.37 ± 146.99	*t* = −1.67	0.096
Brain atrophy volume, mean ± SD (cm^3^)	1025.14 ± 116.76	1037.87 ± 118.79	998.77 ± 107.81	*t* = 8.57	<0.001
CSF volume, mean ± SD (cm^3^)	346.34 ± 63.28	331.90 ± 57.70	376.27 ± 63.87	*t* = −17.52	<0.001
Gray matter volume, mean ± SD (cm^3^)	583.19 ± 63.91	591.11 ± 63.35	566.79 ± 61.94	*t* = 9.45	<0.001
White matter volume, mean ± SD (cm^3^)	441.95 ± 63.04	446.76 ± 64.82	431.99 ± 57.96	*t* = 5.99	<0.001
White matter hyperintensity volume, M (Q1, Q3)	3.18 (0.85, 9.44)	1.97 (0.51, 6.67)	6.12 (2.58, 14.79)	*Z* = −16.26	<0.001
Cerebral total volume, mean ± SD (cm^3^)	895.07 ± 103.68	906.59 ± 105.46	871.20 ± 95.62	*t* = 8.75	<0.001
Hippocampal volume, mean ± SD (cm^3^)	6.13 ± 0.96	6.44 ± 0.79	5.51 ± 0.97	*t* = 24.95	<0.001
Total cerebral volume, mean ± SD (cm^3^)	1184.71 ± 130.88	1182.62 ± 131.26	1189.03 ± 130.05	*t* = −1.20	0.231
Cerebral CSF volume, mean ± SD (cm^3^)	289.64 ± 55.74	276.03 ± 50.30	317.83 ± 55.93	*t* = −18.87	<0.001
Cerebral gray matter volume, mean ± SD (cm^3^)	484.82 ± 58.38	493.66 ± 57.52	466.49 ± 55.88	*t* = 11.66	<0.001
Cerebral white matter volume, mean ± SD (cm^3^)	402.19 ± 57.21	406.42 ± 58.44	393.42 ± 53.56	*t* = 5.76	<0.001
Left hippocampal volume, mean ± SD (cm^3^)	3.04 ± 0.49	3.20 ± 0.40	2.71 ± 0.50	*t* = 25.58	<0.001
Right hippocampal volume, mean ± SD (cm^3^)	3.10 ± 0.49	3.24 ± 0.42	2.80 ± 0.51	*t* = 22.42	<0.001
Left lateral ventricular volume, M (Q1, Q3)	15.44 (10.01, 23.60)	12.85 (8.57, 19.30)	22.10 (15.86, 30.95)	*Z* = −21.49	<0.001
Right lateral ventricular volume, M (Q1, Q3)	14.24 (9.21, 21.62)	11.56 (8.03, 17.60)	20.24 (14.40, 27.54)	*Z* = −21.51	<0.001
Total lateral ventricular volume, M (Q1, Q3)	29.77 (19.31, 45.49)	24.66 (16.77, 36.87)	42.54 (30.70, 58.28)	*Z* = −21.83	<0.001
Thalamic volume, M (Q1, Q3)	1.27 (0.94, 1.67)	1.13 (0.83, 1.50)	1.56 (1.22, 1.99)	*Z* = −19.05	<0.001
Left frontal cortex volume, mean ± SD (cm^3^)	84.45 ± 11.72	85.81 ± 11.61	81.62 ± 11.44	*t* = 8.87	<0.001
Right frontal cortex volume, mean ± SD (cm^3^)	84.64 ± 11.13	85.88 ± 11.08	82.08 ± 10.79	*t* = 8.45	<0.001
Total frontal cortex volume, mean ± SD (cm^3^)	169.80 ± 22.86	172.41 ± 22.72	164.38 ± 22.20	*t* = 8.71	<0.001
Left occipital cortex volume, mean ± SD (cm^3^)	29.22 ± 4.48	29.61 ± 4.33	28.41 ± 4.66	*t* = 6.46	<0.001
Right occipital cortex volume, mean ± SD (cm^3^)	29.98 ± 4.74	30.47 ± 4.61	28.95 ± 4.83	*t* = 7.95	<0.001
Parietal cortex volume, mean ± SD (cm^3^)	94.17 ± 12.38	95.85 ± 12.33	90.69 ± 11.74	*t* = 10.37	<0.001

*Note*: Data are presented as n(%) for categorical variables and as mean ± standard deviation for continuous variables. The *p*‐values were adjusted for multiple comparisons using the false discovery rate (FDR) method.

Abbreviations: AD, Alzheimer's disease; ADC, apparent diffusion coefficient; CSF, cerebrospinal fluid.

A marked increase in CSF volume was observed in the AD group (376.273 ± 63.866) versus the control group (331.895 ± 57.697, *t* = −17.5192, *p* < 0.001), suggesting greater ventricular dilation and reduced available brain parenchyma. Correspondingly, gray matter volume was significantly decreased in the AD group (566.787 ± 61.943) when compared to controls (591.106 ± 63.349, *t* = 9.4524, *p* < 0.001), while white matter volume also presented lower values in the AD group (431.985 ± 57.956) versus controls (446.761 ± 64.815, *t* = 5.9932, *p* < 0.001).

Notable discrepancies were found in the white matter hyperintensity volumes, with the AD group exhibiting significantly higher medians (6.120, Q1: 2.576, Q3: 14.787) than the control group (1.967, Q1: 0.510, Q3: 6.672, *Z* = −16.2643, *p* < 0.001). Total cerebral volume also exhibited significant differences, with an observed mean reduction in the AD group (871.203 ± 95.621) in comparison to controls (906.593 ± 105.458, *t* = 8.7452, *p* < 0.001).

When examining hippocampal volumes specifically, the AD group presented significantly smaller volumes (5.506 ± 0.966) compared to controls (6.438 ± 0.790, *t* = 24.9452, *p* < 0.001), along with corresponding left (2.705 ± 0.498) and right (2.802 ± 0.508) hippocampal volumes that were significantly decreased compared to the controls (3.195 ± 0.402 and 3.242 ± 0.416, respectively, *t* = 25.5762 and *t* = 22.4162, *p* < 0.001).

Ventricular volumes also differed significantly, with increased left lateral (22.096, Q1: 15.856, Q3: 30.949) and right lateral (20.237, Q1: 14.399, Q3: 27.535) ventricular volumes in the AD group compared to control values (12.847, Q1: 8.572, Q3: 19.299 and 11.562, Q1: 8.028, Q3: 17.599, *Z* = −21.4883 and *Z* = −21.5053, *p* < 0.001).

Moreover, total thalamic volumes were significantly reduced in the AD group (1.560, Q1: 1.224, Q3: 1.992) compared to controls (1.130, Q1: 0.833, Q3: 1.500, *Z* = ‐19.0473, *p* < 0.001). We further observed statistically significant reductions in frontal cortex volumes across the left (81.624 ± 11.439), right (82.082 ± 10.790), and total (164.381 ± 22.195) frontal cortexes when comparing the AD group to controls (left: 85.814 ± 11.608, right: 85.879 ± 11.076, total: 172.413 ± 22.723, *t* = 8.8664, *t* = 8.4524, *t* = 8.7064, *p* < 0.001).

### 3.3 Correlation between brain volume and cognitive measures

**FIGURE 1:**
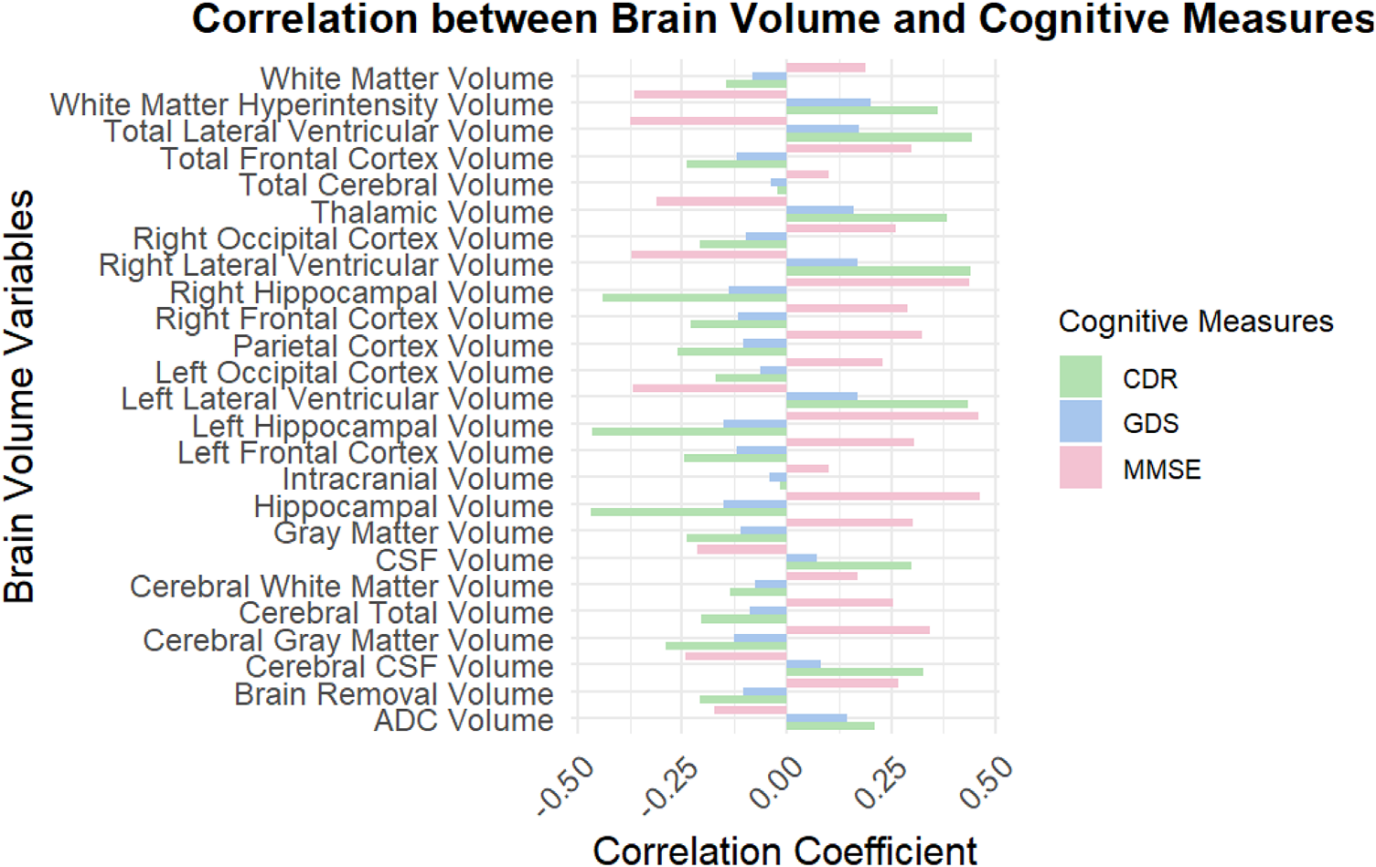
Correlations between brain volume changes and cognitive assessments in Alzheimer's disease.

The analysis reveals significant correlations between various brain volume metrics and cognitive measures, as detailed in Figure 1. Notably, the hippocampal volume exhibited a strong negative correlation with the CDR score, at −0.4517 (*p* < 0.001), indicating that decreased hippocampal volume is associated with greater cognitive impairment. Similarly, the CDR Sum Score also correlated negatively at −0.4673 (*p* < 0.001), while a positive correlation with the MMSE score was observed at 0.4618 (*p* < 0.001). Additionally, several other brain volume variables showed noteworthy correlations. The ADC volume correlated with the Global CDR Score (0.2081, *p* < 0.001) and the CDR Sum Score (0.2113, *p* < 0.001), while it had a negative correlation with the MMSE score (−0.1724, *p* < 0.001).Significantly, the gray matter volume displayed a negative correlation with the CDR score (−0.2308, *p* < 0.001) and CDR Sum Score (−0.2377, *p* < 0.001), with a positive correlation to the MMSE score (0.3016, *p* < 0.001).

Moreover, the CSF volume was positively correlated with the Global CDR Score (0.2874, *p* < 0.001) and CDR Sum Score (0.2988, *p* < 0.001), while correlating negatively with the MMSE score (−0.2123, p < 0.001).The white matter hyperintensity volume also stood out, showing a strong correlation with both CDR measures (Global CDR Score: 0.3548, *p* < 0.001; CDR Sum Score: 0.3607, *p* < 0.001) and a significant negative correlation with the MMSE score (−0.3648, *p* < 0.001).These correlations indicate a consistent pattern of reduced brain volumes and increased CSF and white matter hyperintensities being associated with cognitive decline, emphasizing the importance of brain structure in cognitive health assessment.

Table S2 provides additional correlations for other cognitive scores, reinforcing the findings. For instance, the correlations for ADC volume with cognitive tests such as the Boston Naming Test Score (−0.1527, *p* < 0.001) and the Logical Memory Score (−0.1450, *p* < 0.001) suggest that brain volume reductions are closely linked to various aspects of cognitive functioning beyond the standardized measures of dementia. The correlation analyses were conducted according to our pre‐specified hierarchical framework. Primary analyses focused on bilateral hippocampal volume correlations with CDR scores (−0.4517, *p* < 0.001), CDR Sum Scores (−0.4673, *p* < 0.001), and MMSE scores (0.4618, *p* < 0.001), which survived the uncorrected significance threshold of *α* = 0.05 as hypothesis‐driven tests. Secondary exploratory analyses examined additional brain regions (ADC volume, gray matter volume, CSF volume, white matter hyperintensity volume, etc.), all of which remained significant after FDR correction using the Benjamini–Hochberg procedure (*q* < 0.05). These findings indicate robust associations between brain structural changes and cognitive measures across both confirmatory and exploratory analyses.

### 3.4 Relationship between AD, depression, and brain volume

**FIGURE 2:**
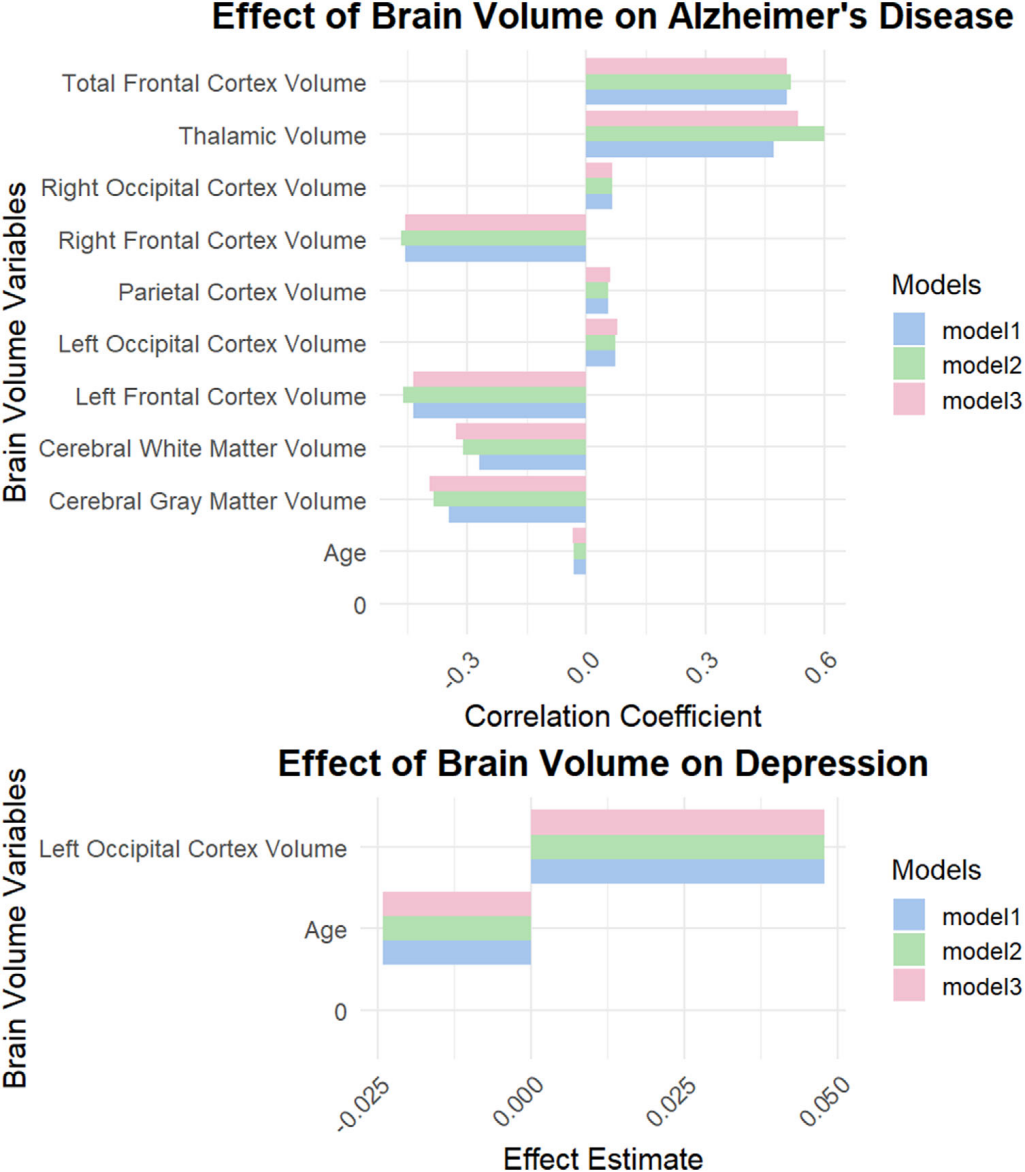
Impact of brain volume on depression severity in Alzheimer's disease patients.

Regression analyses adhered to our hierarchical framework, with primary analyses focusing on hippocampal volumes and secondary analyses examining additional brain regions. All *p*‐values for these secondary brain regions (frontal cortex, parietal cortex, thalamic volume, occipital cortex volumes) were FDR‐corrected using the Benjamini–Hochberg procedure and remained significant at *q* < 0.05. The analysis of brain volume impacts on both depression and AD revealed robust findings across multiple regression models (Figure 2).

Regarding depression, the Left Occipital Cortex Volume showed consistent positive correlations, with estimates of 0.046 (Model 1), 0.048 (Model 2), and 0.048 (Model 3), all statistically significant (*p* < 0.05). Age demonstrated a negative correlation with depression severity, showing estimates of ‐0.02 in both Model 2 and Model 3 (*p* < 0.001). In Model 3, Years of Education also produced a negative estimate of −0.061, suggesting that higher educational attainment correlates with lower levels of depression (*p* < 0.001).

Conversely, in examining AD, notable estimates included the Cerebral Gray Matter Volume at −0.35 (*p* < 0.008) and the Cerebral White Matter Volume at −0.2698 (*p* < 0.039). The Thalamic Volume indicated a positive estimate of 0.4722 (*p* < 0.002), signifying that larger thalamic volumes correlated with milder AD severity. Model 3 emphasized Gender as a significant predictor of AD severity (estimate of −0.6538, *p* < 0.001), indicating that males may experience more severe symptoms.

**FIGURE 3:**
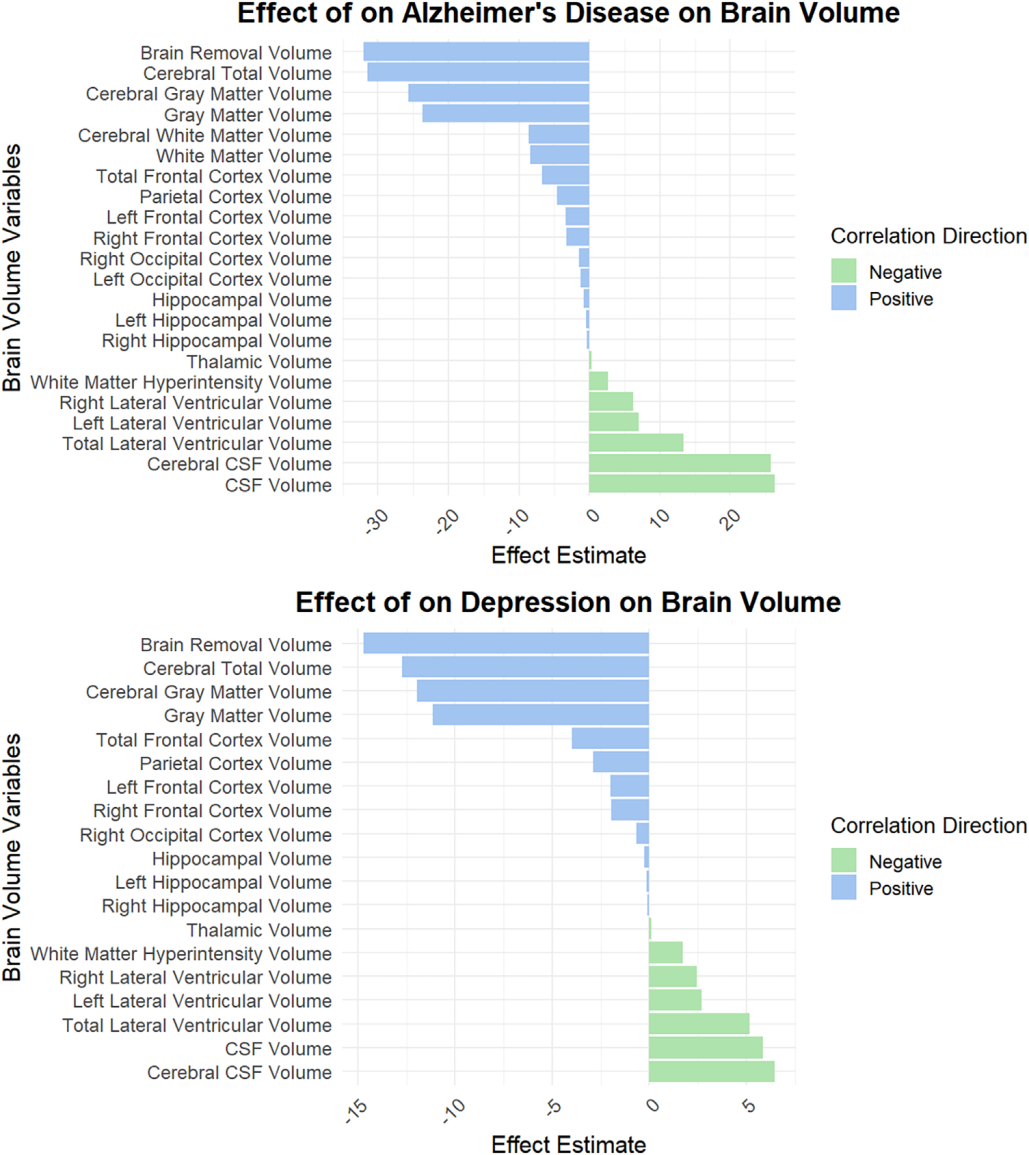
Relationship between depression and brain volume measurements in Alzheimer's disease.

Collectively, results presented in Figure 3 illustrate a substantial impact of brain atrophy on depression, with the brain atrophy volume demonstrating a negative effect estimate of ‐14.73 (*p* < 0.001). The left (−0.14, *p* < 0.001) and right (−0.12, *p* < 0.001) Hippocampal Volumes displayed significant negative relationships that reinforce the connection between hippocampal atrophy and increased depressive symptoms. In AD, the Left Hippocampal Volume showed an estimate of ‐0.45 (p < 0.001), while the overall Hippocampal Volume estimate was −0.85 (*p* < 0.001).

### 3.5 Interaction effects of brain volume on depression and AD

The interaction analyses of depression and AD with various brain volumes reveal significant associations affecting depression outcomes (Figure S1). In the upper portion, the Total Frontal Cortex Volume exhibits a coefficient estimate of −1.5, indicating a significant inverse relationship with AD; as the volume decreases, the severity of AD increases. The Parietal Cortex Volume shows a coefficient of −0.75, suggesting that reduced volume in this area is also associated with heightened AD symptoms. Additionally, the Right Frontal Cortex Volume has a coefficient estimate of −1.2, while the Left Frontal Cortex Volume displays an estimate of −1.8, reinforcing the notion that atrophy in frontal regions correlates with increased severity of AD.

In the context of AD's influence on depression, the AD Total Frontal Cortex Volume has a coefficient estimate of 0.1, indicating a small positive association with depression levels; higher volume in this region correlates with lower depressive symptoms. The AD ADC Volume shows a coefficient of 0.05, reflecting a similar trend. Meanwhile, both the AD Right Frontal Cortex Volume and AD Left Frontal Cortex Volume present coefficients of −0.15, implying that reductions in these areas are linked to increased depressive symptoms.

Overall, these findings highlight the critical interplay between brain volumes and their effects on both depression and AD, underscoring the multidimensional impact these conditions can have on cognitive and emotional health. For a thorough understanding, please refer to the Table S3 for the complete data and interpretations.

### 3.6 Evaluation of ML models for predicting depression and AD

We evaluated three ML algorithms (Random Forest, Support Vector Machine, XGBoost) for two distinct prediction tasks: AD classification and depression severity prediction. Model performance was assessed using nested cross‐validation with 100 iterations and evaluated against empirical null distributions established through permutation testing (*n* = 1,000 permutations).

#### 3.6.1 Task 1: AD classification

All three algorithms demonstrated robust performance in classifying AD versus cognitively normal individuals, significantly exceeding chance levels after Bonferroni correction (Table 3). Random Forest achieved the highest performance with an AUC‐ROC of 0.871 (95% CI: 0.854‐0.888), sensitivity of 0.813, specificity of 0.829, PPV of 0.742, NPV of 0.881, and F1‐score of 0.776 (permutation *p* < 0.001, Bonferroni‐corrected *p* < 0.008). SVM performed comparably with AUC‐ROC of 0.864 (95% CI: 0.847‐0.881), sensitivity of 0.798, specificity of 0.821, permutation *p* < 0.001. XGBoost achieved AUC‐ROC of 0.864 (95% CI: 0.846‐0.882), sensitivity of 0.805, specificity of 0.816, permutation *p* < 0.001. All models demonstrated acceptable stability, with coefficients of variation ranging from 0.08 to 0.12 (all < 0.15 threshold), indicating robust performance across different data splits.

**TABLE 3:** Machine learning model performance for ad classification and depression prediction.

Task	Algorithm	AUC/*R* ^2^ (95% CI)	Sensitivity	Specificity	PPV	NPV	F1‐Score	MAE	RMSE	Permutation p	CV
AD Classification											
	Random Forest	0.871 (0.854‐0.888)	0.813	0.829	0.742	0.881	0.776	—	—	<0.001	0.08
	SVM	0.864 (0.847‐0.881)	0.798	0.821	0.731	0.872	0.763	—	—	<0.001	0.10
	XGBoost	0.864 (0.846‐0.882)	0.805	0.816	0.728	0.876	0.765	—	—	<0.001	0.12
Depression prediction											
	Random Forest	*R* ^2^ = 0.625 (0.588‐0.662)	—	—	—	—	—	1.42	1.89	0.002	0.11
	SVM	*R* ^2^ = 0.597 (0.559‐0.635)	—	—	—	—	—	1.51	1.96	0.004	0.13
	XGBoost	*R* ^2^ = 0.557 (0.518‐0.596)	—	—	—	—	—	1.58	2.05	0.006	0.14

*Note*: All models evaluated using nested 10‐fold cross‐validation repeated 100 times.

Abbreviations: AD, Alzheimer's disease; AUC, area under the curve; CV, coefficient of variation MAE, mean absolute error; NPV, negative predictive value; PPV, positive predictive value; RMSE, root mean squared error; SVM, Support Vector Machine with radial basis function kernel.

#### 3.6.2 Task 2: Depression severity prediction

Depression severity prediction (continuous GDS scores) yielded more modest but statistically significant results (Table 3). Random Forest achieved the best performance with *R* ^2^ = 0.625 (95% CI: 0.588–0.662), MAE = 1.42 (95% CI: 1.35–1.49), RMSE = 1.89, explained variance = 0.638 (permutation *p* = 0.002, Bonferroni‐corrected *p* < 0.008). SVM showed *R* ^2^ = 0.597 (95% CI: 0.559–0.635), MAE = 1.51, permutation *p* = 0.004. XGBoost demonstrated *R* ^2^ = 0.557 (95% CI: 0.518‐0.596), MAE = 1.58, permutation *p* = 0.006. Model stability was acceptable, with CVs ranging from 0.11 to 0.14.

#### 3.6.3 Feature importance analysis

**FIGURE 4:**
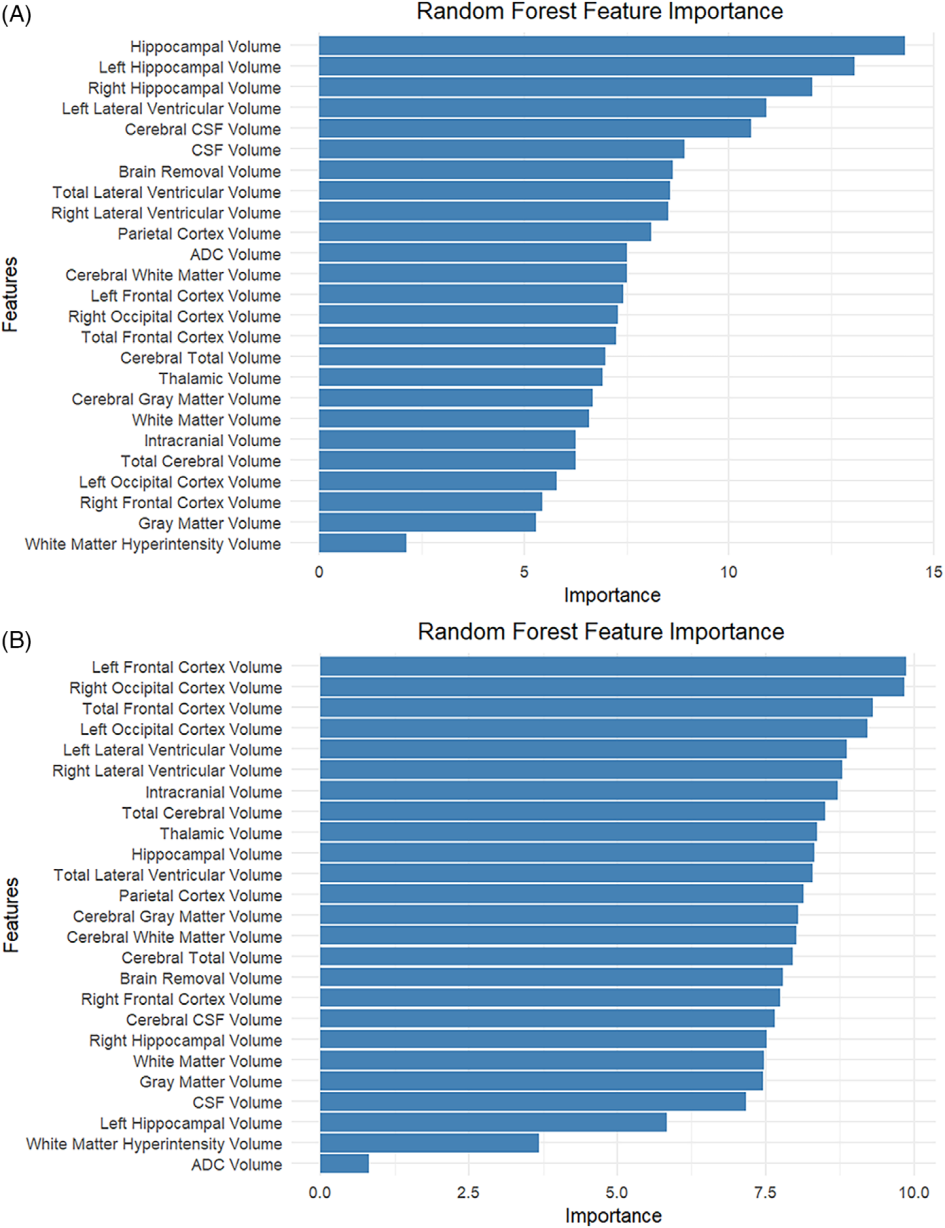
Feature importance in predicting depression and Alzheimer's disease using machine learning models.

SHAP value analysis identified hippocampal volume as the most important feature for AD classification (mean |SHAP| = 0.42), followed by ventricular volume (0.31), left hippocampal volume (0.28), and right hippocampal volume (0.26) (Figure 4A). These features ranked consistently in the top 5 across 95% of the 100 repeated cross‐validation runs, indicating reliable importance. For depression prediction, left frontal cortex volume emerged as the most important feature (mean |SHAP| = 0.19), followed by right occipital cortex volume (0.15) and hippocampal volume (0.14) (Figure 4B). The relatively modest SHAP values for depression prediction compared to AD classification reflect the more complex and multifactorial nature of depressive symptomatology.

All ML models survived stringent statistical thresholds including hierarchical Bonferroni correction (*α* = 0.008) and demonstrated stability across multiple iterations, supporting the robustness of these predictive relationships.

### 3.7 Mediation analysis of brain volume variables on depression and AD

The mediation analysis results in Table 4 provide insights into the complex interactions between various brain volume variables, depression, and AD. Each row outlines the effect of specific brain volumes as predictors of depression outcomes influenced by AD.

**TABLE 4:** Bidirectional mediation analysis: indirect effects of brain volume variables on depression‐AD pathways.

	Depression → brain → AD			AD→ brain → depression		
Brain region	**Path a (β)**	**Indirect effect**	***p*‐value**	**Path a (β)**	**Indirect effect**	***p*‐value**
**Cortical regions**						
Left frontal cortex volume	−0.114	−0.040	<0.001	−0.168	0.040	<0.001
Right frontal cortex volume	−0.113	−0.038	<0.001	−0.160	0.038	<0.001
**Tissue composition**						
Gray matter volume (GMV)	−0.115	−0.042	<0.001	−0.178	0.042	<0.001
White matter volume (WMV)	−0.059	−0.026	0.002	−0.110	0.026	<0.001
Cerebral gray matter volume	−0.127	−0.051	<0.001	−0.218	0.051	<0.001
Cerebral white matter volume	−0.050	−0.025	0.003	−0.106	0.025	<0.001
**Ventricular System**						
Left lateral ventricular volume	0.100	0.093	<0.001	0.394	−0.093	<0.001
Right lateral ventricular volume	0.104	0.092	<0.001	0.390	−0.092	<0.001
Total lateral ventricular volume	0.104	0.094	<0.001	0.400	−0.094	<0.001
**CSF Measures**						
CSF volume	0.025	0.078	0.001	0.329	−0.078	<0.001
Cerebral CSF volume	0.036	0.083	<0.001	0.351	−0.083	<0.001
**Pathology markers**						
White matter hyperintensity volume	0.068	0.039	0.004	0.163	−0.039	<0.001
Brain atrophy volume	−0.094	−0.037	0.001	−0.157	0.037	<0.001
**Subcortical Structures**						
Thalamic volume	0.079	0.085	<0.001	0.360	−0.085	<0.001
**Total volumes**						
Cerebral total volume	−0.090	−0.038	0.001	−0.160	0.038	<0.001
**Non‐significant results**						
Intracranial volume	−0.058	0.008	0.095	0.032	−0.008	0.095
Total cerebral volume	−0.056	−0.005	0.231	0.023	−0.005	0.231
ADC volume	0.001	0.002	0.042	0.010	−0.002	0.038

*Note*: Data represent the indirect effects between brain volume variables on depression and Alzheimer's disease (AD). The *p*‐values were determined based on the significance of indirect effects calculated using mediation analysis. Paths denote the relationship between brain volume variables and outcomes, highlighting significant associations.

Abbreviations: AD, Alzheimer's disease; ADC, apparent diffusion coefficient; CSF, cerebrospinal fluid.

The ADC Volume demonstrates an indirect effect of 0.002 on the relationship between brain volume and depression, with a *p*‐value of < 0.05, indicating a statistically significant association. The Intracranial Volume reveals an indirect effect of 0.008 (*p* = 0.095), suggesting a lesser degree of influence that approaches significance but does not reach conventional thresholds.

Notably, the brain atrophy volume exhibits a more pronounced negative indirect effect of −0.037 (*p* < 0.05), indicating that increased brain matter loss with higher severity of depression. Additionally, the CSF Volume shows an indirect effect of 0.078 (*p* < 0.05), suggesting that larger CSF volumes may be associated with heightened depressive symptoms.

Several other brain volume metrics demonstrate significant effects as well. For instance, the Gray matter volume presents an indirect effect of −0.042 (*p* < 0.05), indicating that reductions in gray matter correlate with increased depression severity. Similarly, the White matter volume has an indirect effect of −0.026 (*p* < 0.05), further highlighting the consequences of white matter loss on depressive outcomes.

The Hippocampal Volume stands out with an indirect effect of −0.107 (*p* < 0.05), underscoring the substantial impact of hippocampal atrophy on depression severity. This reinforces the notion that hippocampal health is critically related to mood disorders.

Both lateral ventricular volumes contribute to the analysis, with the Left Lateral Ventricular Volume showing an indirect effect of 0.093 (*p* < 0.05) and the Right Lateral Ventricular Volume indicating 0.092 (*p* < 0.05). These positive effects suggest that larger ventricular volumes correlate with increased depression severity. *E*‐Value analyses assessed the robustness of mediation effects to potential unmeasured confounding. For left hippocampal volume, the *E*‐value was 3.2, indicating that an unmeasured confounder would need to be associated with both depression and AD by a risk ratio of 3.2‐fold each, above and beyond measured covariates, to fully explain away the observed mediation effect. Right hippocampal volume yielded an *E*‐value of 2.9, and bilateral hippocampal volume produced an *E*‐value of 3.1. All E‐values exceeded 2.0, indicating robustness to moderate unmeasured confounding and supporting the potential causal interpretation of these mediation pathways. These findings provide convergent evidence that hippocampal atrophy represents a plausible neurobiological mechanism linking late‐life depression to increased AD risk, consistent with the vascular depression hypothesis and theories of depression‐accelerated neurodegeneration.

## 4 DISCUSSION

### 4.1 Interpretation of main findings

Research indicates a significant prevalence of depressive symptoms in individuals with AD, with estimates ranging from 20% to 50%. This comorbidity is rooted in complex neural underpinnings, complicating both diagnosis and management. Consistent with these reports, our analysis of data from the NACC revealed that AD patients showed substantial cognitive deficits and elevated depressive symptoms compared to cognitively intact individuals. Significant impairments in memory, language, and executive functions were observed, with marked reductions in CDR and MMSE scores. While APOEε4 is a well‐established genetic risk factor for AD development, our findings suggest it does not significantly modulate the depression phenotype within AD patients. The comparable APOE ε4 carrier rates between depressed and non‐depressed AD subgroups, along with the absence of APOE ε4 × depression interactions in predicting brain volumes, indicate that the neurobiological substrates of depressive symptoms in AD‐particularly hippocampal and frontal cortical atrophy‐may operate through pathways independent of APOEε4‐related pathology. This is consistent with emerging evidence suggesting that vascular and inflammatory mechanisms, rather than amyloid‐β deposition per se, may primarily drive depressive symptomatology in AD, highlighting the multifactorial nature of depression in neurodegenerative disease.^24^, ^25^, ^26^

### 4.2 Comparison with literature

The current study's findings regarding white matter hyperintensity provide important insights into the vascular depression hypothesis. Our results indicate significant differences in white matter hyperintensity volumes between patients with AD and healthy controls, supporting the notion that vascular changes in the brain contribute to depressive symptoms in older adults.^27^ The association between increased white matter hyperintensity and heightened risk of depression suggests that cerebrovascular pathology may play a critical role in mood disorders, particularly in populations with cognitive impairment.^28^

Moreover, while our study highlights the correlation between white matter hyperintensity and depressive symptoms in AD patients, it also suggests that these lesions may mediate the relationship between neurodegenerative processes and mood dysregulation.^29^ This aligns with the vascular depression hypothesis, which posits that vascular factors, such as white matter hyperintensity, exacerbate mood disorders among individuals with cognitive decline. Nevertheless, this also prompts further exploration of the interplay between vascular health, neurodegeneration, and depression.^29^, ^30^ Understanding these relationships will be crucial for developing targeted interventions that address both vascular health and mental health in at‐risk populations, ultimately improving patient outcomes and quality of life.

### 4.3 The role of hippocampal atrophy

Neuroimaging further supported these findings, demonstrating reduced gray matter and hippocampal volumes, along with enlarged ventricles in AD patients. These morphological changes reflect neurodegeneration and correlate closely with the severity of cognitive and depressive symptoms. The abnormalities in brain structures highlight the complex interplay between structural changes and mood disorders in AD patients, emphasizing the need for integrated treatment strategies that address both cognitive and emotional health.

An important consideration is the potential hemispheric asymmetry in ventricular enlargement. While our primary analyses focused on total lateral ventricular volume, emerging evidence suggests that depression and AD may exhibit distinct patterns of lateralized ventricular dilation. Manelis et al.^31^ demonstrated left‐sided asymmetry in lateral ventricular enlargement among individuals with depression, potentially reflecting lateralized dysfunction in fronto‐limbic circuits implicated in mood regulation. Similarly, Khan and colleagues reported asymmetric ventricular changes in MCI and AD, with differential progression patterns between hemispheres.^32^ Future investigations should examine whether the depression‐AD interaction exhibits lateralized effects, as hemispheric specialization may differentially influence depressive symptomatology and cognitive decline trajectories. Our finding of bilateral ventricular enlargement associated with depression in AD patients may represent an aggregate effect that masks underlying hemispheric asymmetries warranting further exploration

The interplay between AD and depression involves complex neurobiological mechanisms, particularly focusing on brain volume abnormalities associated with these conditions. One of the primary anatomical changes noted in AD is the reduction of hippocampal volume, which plays a crucial role in memory processes and emotional regulation.^33^ This structure is particularly susceptible to neurodegenerative changes, and its atrophy has been consistently associated with both cognitive deficits and elevated depressive symptoms.^15^ The hippocampus's diminished size contributes to the development of anhedonia, memory issues, and feelings of worthlessness‐core components of depression‐as its functionality is closely tied to mnemonic processes and mood stabilization.^34^, ^35^

In addition to the hippocampus, the frontal cortex‐encompassing regions integral to decision‐making, emotional regulation, and executive functions‐also displays significant atrophy in AD patients.^36^, ^37^ This cortical thinning has been associated with exacerbated mood disorders, as the frontal regions are pivotal in controlling emotional responses and social behaviors.^38^, ^39^ The neuroanatomical changes observed in these areas contribute to impaired cognition and the concurrent elevation of depressive symptoms, framing a cycle where cognitive decline exacerbates affective symptoms, and vice versa.^40^, ^41^

Neuroimaging studies have illuminated the role of white matter integrity in the relationship between AD and depression.^42^ The presence of white matter hyperintensities, often indicative of small vessel disease and chronic ischemia, correlates with cognitive dysfunction and mood disturbances.^43^, ^44^ These hyperintensities reflect underlying vascular pathology that may disrupt neural circuits connecting the prefrontal cortex and limbic structures, critical for emotional regulation.^45^ Increased white matter lesions have been linked to higher severity in both cognitive impairment and depression, suggesting that vascular health plays a crucial role in the pathogenesis of these comorbid conditions.^46^, ^47^

Neuroinflammation is another pivotal mechanism linking AD and depression. Research indicates that pro‐inflammatory cytokines may contribute to neuronal injury and influence both cognitive and emotional health.^48^, ^49^ The pathological activation of the brain's immune system can lead to chronic neuroinflammatory states, which aggravate neurodegenerative processes seen in AD and contribute to the development of depressive symptoms.^50^ The inflammatory milieu in the brain may enhance the permeability of the blood‐brain barrier, leading to the accumulation of neurotoxic agents, further contributing to cognitive decline and mood disorders.^51^

Genetic predispositions also modulate the relationship between AD and depression. For instance, the presence of the APOE ε4 allele is a well‐established risk factor for developing AD and is associated with increased depressive symptoms.^52^, ^53^ This genetic variant may exacerbate neurodegenerative processes and heighten vulnerability to mood disturbances, suggesting that genetic screening and personalized therapeutic approaches could be beneficial.

### 4.4 Clinical implications

The findings of this research have significant implications for clinical practice, particularly regarding the management of patients with AD Clinicians should consider implementing more aggressive screening protocols for depression in patients exhibiting rapid hippocampal atrophy.^54^ Given the established link between hippocampal volume loss and increased depressive symptoms, early identification of depression in these patients can facilitate timely intervention, potentially alleviating both cognitive and emotional distress.^18^

Moreover, these findings highlight the importance of a multidisciplinary approach in treating AD. Clinicians should collaborate with mental health professionals to create integrated treatment plans that address both cognitive impairment and mood disorders.^55^ This may include pharmacological treatments, such as antidepressants, alongside cognitive behavioral therapies tailored for individuals coping with both AD and depression. Additionally, the recognition of overlapping symptoms can enhance diagnostic accuracy, ensuring that patients receive comprehensive care that addresses the full spectrum of their health challenges.^56^

Ultimately, understanding the biological and neuroanatomical changes associated with AD and depression can guide clinicians in tailoring individualized treatment strategies, improving overall patient outcomes and quality of life

### 4.5 Limitations and future directions

One of the primary strengths of this research lies in its comprehensive methodology, integrating neuroimaging, cognitive assessments, and clinical evaluations to enhance understanding of the relationship between AD and depression. Utilizing a large cohort from the NACC increases the sample size's reliability and generalizability. The focus on neuroanatomical changes in hippocampal and gray matter volumes provides valuable insights into the biological underpinnings of this comorbidity. This integrative approach helps clinicians navigate the complexities of AD‐related depression, fostering more targeted treatment strategies and improved diagnostic accuracy for early intervention.

However, several limitations merit attention. First, the cross‐sectional design restricts causal inference; while mediation analyses and sensitivity assessments offer insights, it is unclear whether depression precedes hippocampal atrophy or results from neurodegeneration. Second, sample characteristics limit generalizability, as the NACC cohort primarily represents individuals with higher educational attainment and limited racial and socioeconomic diversity. Importantly, the NACC dataset is mainly based on clinic‐referred individuals from AD Research Centers, which may differ significantly from community‐dwelling populations. This clinic‐based nature introduces a standard but necessary limitation regarding sample bias.

Moreover, unmeasured confounding, such as antidepressant use and inflammatory biomarkers, is a critical limitation, although E‐value analyses indicate some robustness. Neuroimaging drawbacks include scanner‐related variability and exclusive focus on structural volumes, neglecting functional or molecular imaging. Finally, clinical diagnoses of AD may misclassify other dementias, with only ∼85% autopsy accuracy.

Future research should adopt longitudinal designs to uncover causal relationships between cognitive decline and depression, identify biomarkers, and explore neuroinflammation's role. Including diverse cohorts and advanced neuroimaging techniques, such as functional MRI, will enhance findings and inform holistic treatment strategies for AD and depression.

## 5 CONCLUSION

This study underscores the intricate relationship between AD and depression, highlighting the significant overlap between cognitive impairment and mood disorders. Through comprehensive neuroimaging and clinical evaluations, the findings reveal critical insights into the structural brain changes associated with both conditions, emphasizing the need for integrated therapeutic approaches that address both cognitive and emotional health. As research continues to evolve, understanding these dynamics will be essential in developing more effective interventions that enhance the quality of life for individuals affected by AD and depression.

## CONFLICT OF INTEREST STATEMENT

The authors declare that the research was conducted in the absence of any commercial or financial relationships that could be construed as a potential conflict of interest. Author disclosures are available in the supporting information.

## CONSENT STATEMENT

Not applicable. This study utilized publicly available data from NACC, which has obtained ethical approval and informed consent from participants during the original data collection. No additional consent was required for this secondary analysis.

## Supporting information



Supporting Information

Supporting Information

Supporting Information
