# Supplementary material for: Association between brain volume and depression in Alzheimer's disease: Neuroimaging insights
Source: Alzheimers Dement. 2026 Jan 29;22(2):e71120. doi: 10.1002/alz.71120 (PMC12854940; doi:10.1002/alz.71120)

Supplementary materials Fig.1: Interaction Effects of Brain Volume on Depression and Alzheimer's Disease

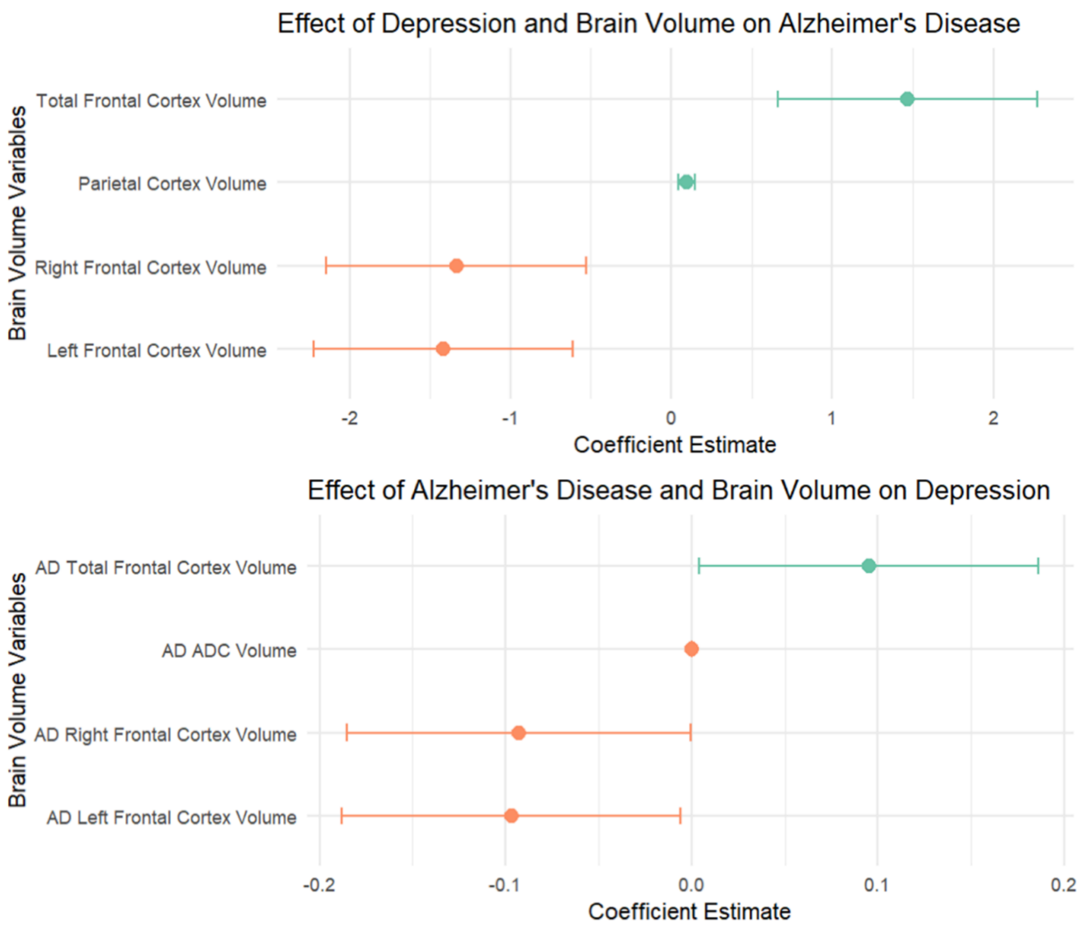

Supplement: Supplementary file 1 — Supporting Information [file ALZ-22-e71120-s001.pdf]
